# Defying the odds: A rare case of primary breast double-hit lymphoma with remarkable remission

**DOI:** 10.1177/2050313X261417125

**Published:** 2026-03-20

**Authors:** Lina Z. Alsaket, Sarah A. Elkourashy, Jouhar Kolleri, Amal M. Alobadli

**Affiliations:** 1 Breast Care Unit, Clinical Imaging Department, Hamad Medical Corporation, Doha, Qatar; 2 Department of Hematology and Bone Marrow Transplant, National Center for Cancer Care and Research, Hamad Medical Corporation, Doha, Qatar; 3 Weill Cornell Medicine – Qatar (WCM-Q), Doha, Qatar; 4 Clinical Imaging Department, Hamad Medical Corporation, Doha, Qatar

**Keywords:** primary breast double-hit lymphoma, Breast mass, PET–CT, DA-EPOCH

## Abstract

Malignant lymphoma in the breast is rare, with primary breast lymphoma and secondary breast lymphoma being the two subtypes. Primary breast lymphoma presents as a fast-growing, painless, palpable mass and is less frequent due to limited lymphoid tissue in the breast. Primary breast double-hit lymphoma is a very rare, highly aggressive malignancy that presents a great challenge regarding proper diagnosis and optimal treatment. Our case involved high-grade B-cell lymphoma with MYC and BCL2 rearrangement (double hit), treated with dose-adjusted etoposide, prednisone, vincristine, cyclophosphamide, and doxorubicin chemotherapy, resulting in complete resolution.

## Introduction

Malignant lymphoma rarely arises in the breast and comprises two subtypes, primary breast lymphoma (PBL) and secondary breast lymphoma (SBL). PBL is defined as lymphoma confined to the breast without evidence of systemic disease, while SBL occurs when systemic lymphoma involves the breast. Due to the limited lymphoid tissue in the breast, PBL is less common and often presents as a rapidly growing, palpable mass mimicking breast carcinoma. Histological examination is crucial for accurate diagnosis, and recent studies have identified a subset of high-grade B-cell lymphomas with MYC and BCL2 rearrangement, termed “double-hit lymphoma” (DHL). There are no standardized guidelines for the treatment of primary breast DHL (PB-DHL). Here, we present a case of high-grade B-cell lymphoma with MYC and BCL2 rearrangement of germinal center cell type, successfully managed with regimen dose-adjusted etoposide, prednisone, vincristine, cyclophosphamide, and doxorubicin (DA-EPOCH) chemotherapy.

## Case presentation

A 45-year-old Tunisian patient, a mother of three and a medical staff nurse, presented to the outpatient breast surgery clinic in May 2022 with a palpable lump in her right breast for several days. She was otherwise asymptomatic, with no fever, weight loss, night sweats, or other B symptoms, and had no family history of breast cancer or lymphoma. On physical examination, a 2 cm palpable lump was noted at the 9:00 position in the right breast with mild tenderness, while the examination of the left breast was unremarkable.

Basic laboratory tests, including complete blood counts, kidney, and liver functions, were acceptable. Viral serology yielded negatives for HIV, hepatitis B, C, and HTLV antibody. PCR for CMV, EBV, and adenovirus all returned negative results. TB Quantiferon was negative as well. Lactate dehydrogenase was 190 U/L (135–214).

**Figure 1.**
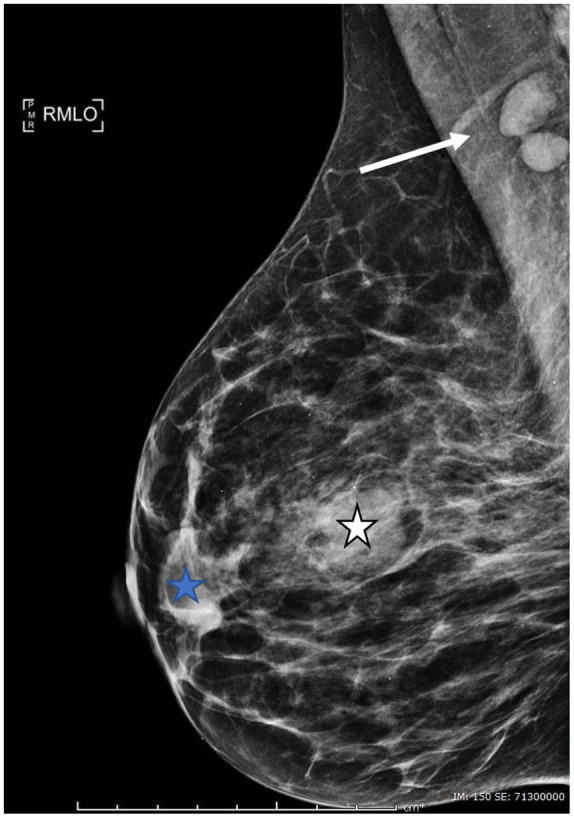
Right-sided mammogram MLO (Mediolateral Oblique) view, showed a rounded isodense mass (white asterisk) with partially obscured margin at 9:00 and another similar smaller one at 9:00 retroareolar (blue asterisk), with prominent dense right axillary lymph nodes (arrow).

**Figure 2.**
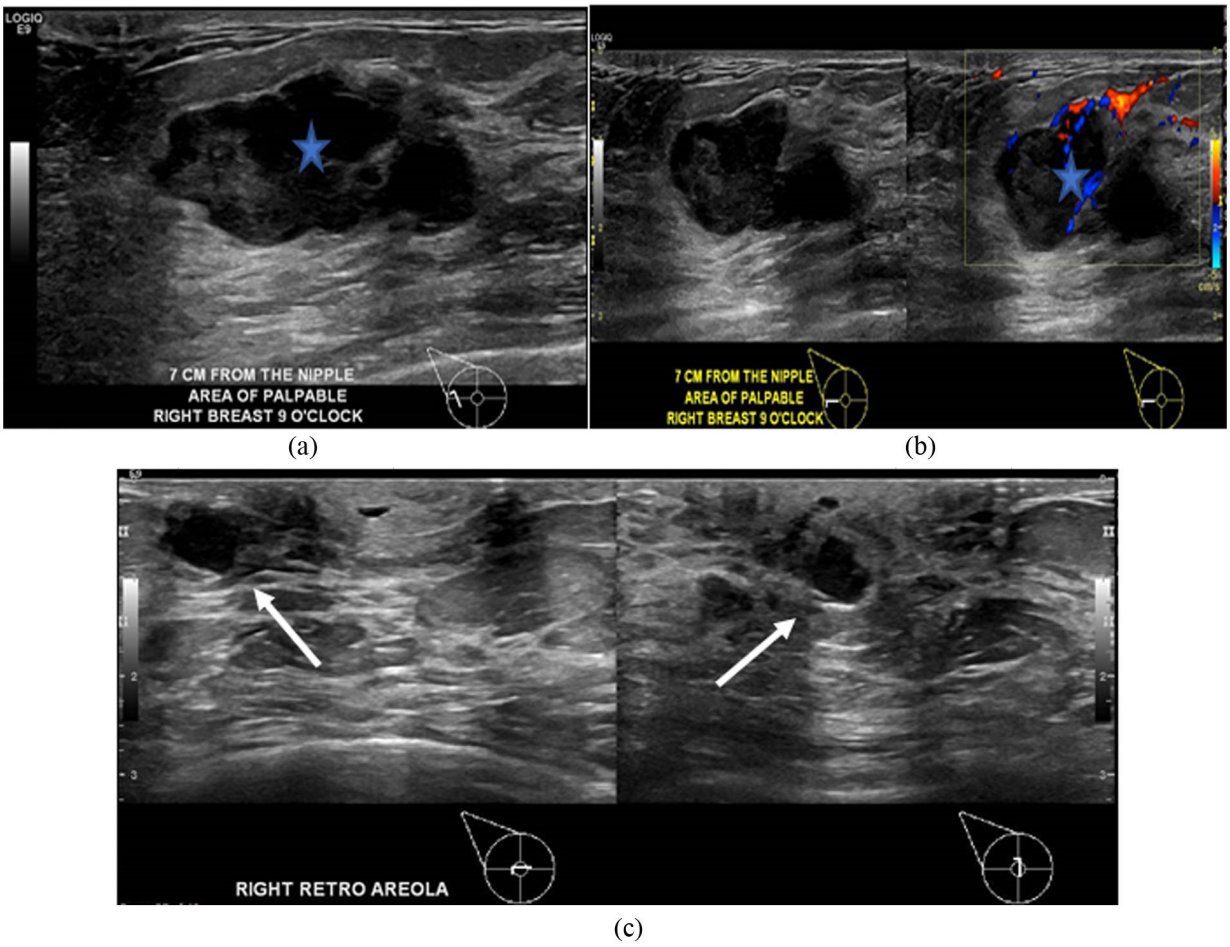
Right breast ultrasound showed two hypoechoic oval masses with lobulated margins and an intra-cystic component extending to the nipple with possible intraductal position of the masses. (a) Showed a hypoechoic mass (asterisk) with lobulated margins at 9:00, 7 cm from nipple. (b) Showed mass with significant intralesional vascularity (asterisk). (c) Showing the extension of the mass toward the base of the nipple with possible dilated ducts (arrow).

Mammogram and breast ultrasound (Figures 1 and 2) were ordered accordingly and done on the same day of referral from the breast surgery clinic, revealing masses with the largest measuring 3 cm with a cystic component at 9:00 in the right breast, near the base of the nipple, and associated with ipsilateral enlarging axillary lymph nodes. The initial diagnosis was consistent with papillary lesions that resembled intraductal masses, raising concerns of BIRADS 4B, which warrant biopsy.

Ultrasound-guided core biopsy with a 14-gauge needle was performed for two masses in the right breast at 9:00, and retro areolar 9:00. Tissue marker clips were deployed, biopsy of the right axillary lymph node was also performed.

**Figure 3.**
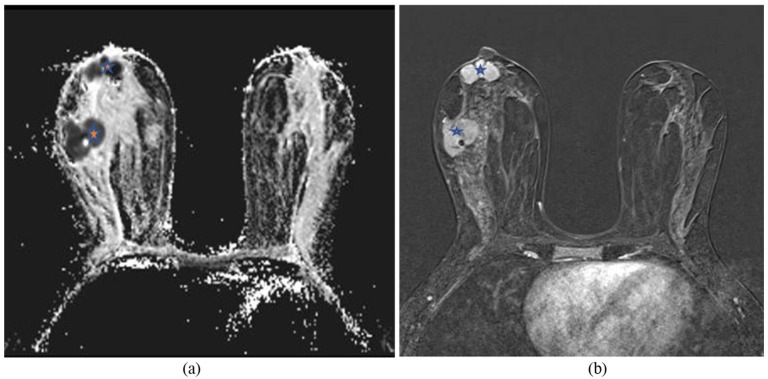
Breast MRI with IV contrast showed a suspicious mass in the right breast with a tissue marker (gel mark) in the dominant mass. (a) Axial ADC image showed hypointense signal masses with low ADC values (0.3; asterisk). (b) T1 Fat-sat subtracted axial image with IV contrast showed an avidly enhancing mass at 9:00 and 9:00 retroareolar extending to the base of the nipple with no signs of nipple invasion (asterisk).

Breast MRI was done and showed multifocal (at least four) multilobulated masses with restriction on DWI (Diffusion Weighted Imaging) and low ADC (Apparent Diffusion Coefficient) values (mean 0.3; Figure (3(a)) shows the kinetic curve type III of enhancement on post-contrast images (Figure 3(b)). Some of these masses showed central necrosis, as well as prominent right axillary lymph nodes (level I), with the left breast appearing unremarkable.

Histopathology analysis of the right breast mass and right axillary lymph node revealed large B-cell lymphoma, with a Ki67 index of 95%, triple expressor, classified as the germinal center cell type.

**Figure 4.**
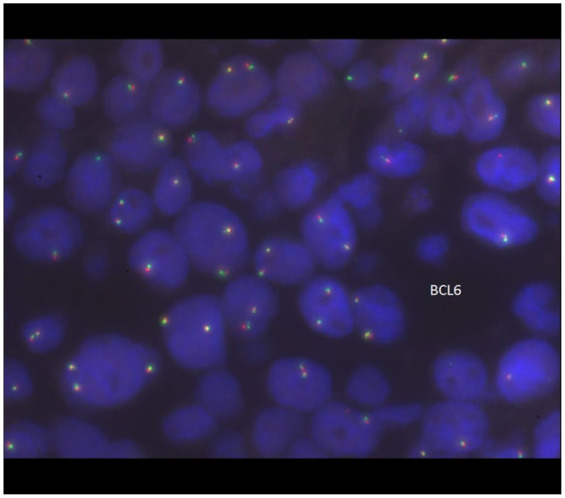
FISH of the lymphoma cells showed the signal pattern for the BCL6 probe was within normal limits with no evidence of rearrangement. FISH: fluorescence in situ hybridization.

**Figure 5.**
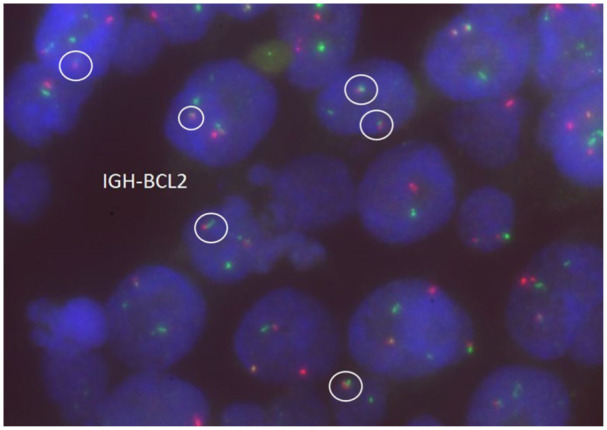
FISH of the *IGH–BCL2* was performed on interphase cells, and fusion in lymphoma cells is demonstrated (white circles). FISH: fluorescence in situ hybridization.

**Figure 6.**
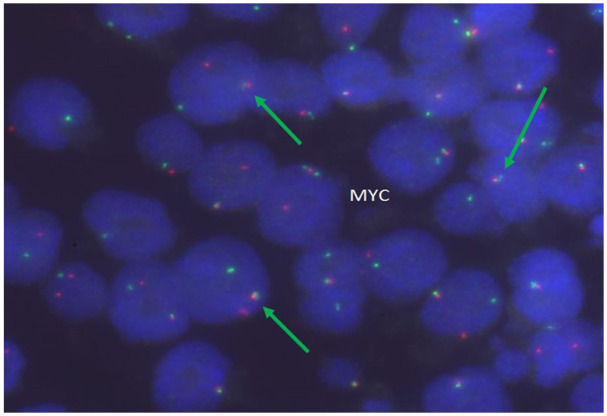
FISH of the *IGH–MYC* fusion was performed on interphase cells; the fusion of *IGH* and *MYC* genes in lymphoma cells was noted (green arrows). FISH: fluorescence in situ hybridization.

Subsequently, fluorescence in situ hybridization (FISH) was performed on the slides using probes for BCL6 at 3q27, BCL2/IGH t (14,18) (q32; q21), and MYC at 8q24. The signal pattern for the BCL6 probe was within normal limits, with no evidence of rearrangement (Figure 4). The FISH analysis of IGH/BCL2 (Figure 5) probe showed a signal pattern consistent with rearrangement in 96% nuclei, with additional signals for IGH indicating another translocation of IGH with another partner, identified as t (8:14), MYC/IGH. The FISH analysis of the MYC probe was consistent with the presence of a MYC rearrangement in 96% nuclei (Figure 6).

Epstein–Barr encoding region in situ hybridization, kappa, and lambda by in situ hybridization are negative.

High-grade B-cell lymphoma with MYC and BCL2 rearrangement according to the fifth edition of the World Health Organization classification of lymphoid neoplasms 2022.^1,2^

The diagnosis was updated as high-grade B-cell lymphoma with MYC and BCL2 rearrangement (DHL), germinal center cell type.

**Figure 7.**
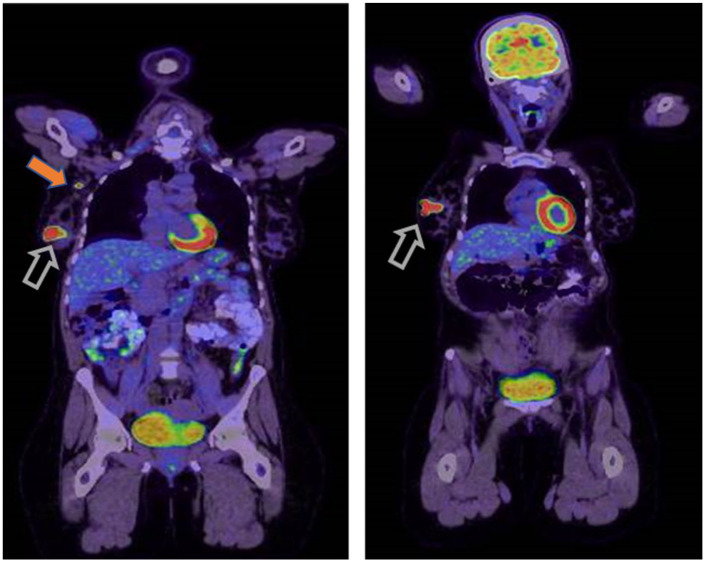
PET–CT scan showed two lobulated hypermetabolic masses in the lower quadrant of the right breast (open arrows) and an intensely hypermetabolic lymph node in the right axilla (block arrow). PET–CT: positron emission tomography–computed tomography.

To complete her staging work up, a positron emission tomography–computed tomography (PET–CT) scan (Figure 7) was requested and showed two lobulated hypermetabolic masses in the outer quadrant of right breast measuring between 2.5 and 3 cm in largest diameter (SUVmax 18.3), as well as an intensely hypermetabolic 1 cm short diameter lymph node in the right axilla. The remainder of the scan showed no remarkable findings. Bone marrow examination was done and showed no evidence of involvement by lymphoma. FISH was performed on bone marrow using probes for IGH/BCL2 and IGH/MYC/CEP8, and the results were normal. An initial lumbar puncture ruled out central nervous system (CNS) involvement.

The patient was staged as Stage II–E DHL. Since DHL patients are at high risk of CNS relapses, which necessitate an intensification of the chemotherapy protocol and the addition of a CNS prophylaxis regimen, the lymphoma multidisciplinary meeting panel recommended six cycles of chemotherapy. CNS prophylaxis in PB cases is recommended due to higher CNS recurrence rates in PB diffuse large B-cell lymphoma (DLBCL).

She received six cycles of chemotherapy with DA-EPOCH-R every 21 days; the first dose was introduced through a Hickman catheter into the right internal jugular vein. This regimen includes DA-EPOCH and rituximab (DA-EPOCH-R). CNS prophylaxis includes four cycles of intrathecal chemotherapy and two cycles of intravenous high-dose methotrexate. The treatment course was uneventful, with no major complications apart from febrile neutropenia.

**Figure 8.**
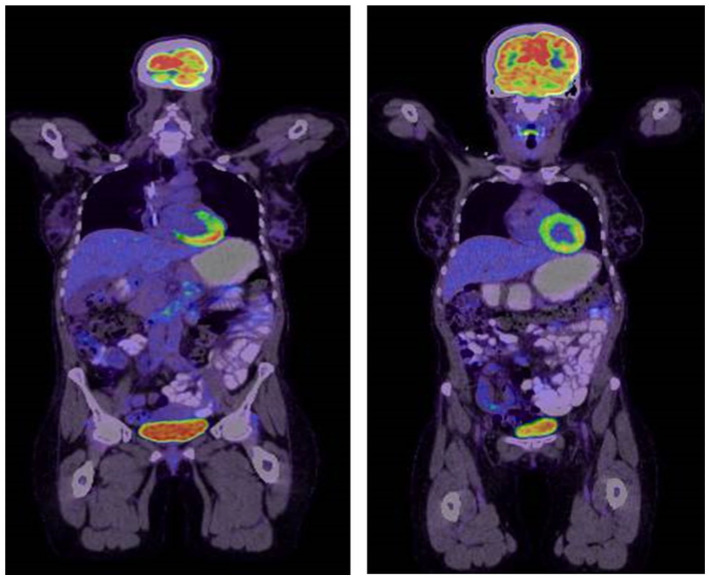
PET–CT scan showed complete response with no suspicious uptake, suggestive of complete response. PET–CT: positron emission tomography–computed tomography.

We followed the patient with a PET–CT scan after four cycles of DA-EPOCH-R, which showed a favorable response, and then at the end of treatment after completing six cycles, another PET–CT confirmed complete metabolic response (Figure 8).

With the intensification of chemotherapy, we decided to omit breast radiotherapy to minimize the risk of late breast cancer and secondary malignancy. This decision aligned with the patient’s preference as well.

Currently, the patient remains symptom-free 3 years after completing her treatment and is being kept under surveillance.

## Discussion

It is uncommon for malignant lymphoma to develop in the breast. PBL and SBL are two subtypes of breast lymphoma. The presence of breast lymphoma without evidence of systemic disease is known as PBL. When systemic lymphoma concurrently or subsequently affects the breast, it is called SBL.^1,2^ PBL makes up about 2% of all extranodal non-Hodgkin lymphomas, and the lifetime risk of non-Hodgkin lymphoma for women is roughly 1.8%.^3^

The classic lymphoma B symptoms, such as fever, weight loss, and night sweats, are quite uncommon in PBL.^4^ PBLs are less frequent than lymphomas of other organs because the breast has less lymphoid tissue compared to the gastrointestinal tract or the lung. Typically, PBL mimics breast carcinoma and usually presents with fast growing, mobile, painless, palpable, solitary mass, with or without ipsilateral axillary lymphadenopathy. It is most predominant in women in their fifth to sixth decade of life. The right breast is the most involved site.^1,2^

Radiographic findings tend to be nonspecific as mammographic imaging typically shows a solitary, noncalcified, well circumscribed, or indistinctly marginated high-density mass. Ultrasound imaging demonstrates focal or diffuse involvement of hypoechoic lesions with poorly defined boundaries, implying enhanced vascularity, whereas MRI may be more sensitive in detecting multicentric lesions.^5^ On dynamic MRI, PBL often shows fast early-phase enhancement reflecting hypervascularity. In addition, diffusion-weighted images, which reflect cell density, demonstrate restricted diffusion.^4^

Excisional biopsy and core biopsy are the preferred techniques for adequate tissue acquisition; the latter is preferred due to its minimally invasive nature. An umbrella term for morphologically aggressive B-cell lymphomas with MYC rearrangement and additional rearrangement of other recognized oncogenes is “DHL”. Aukema et al. analyzed the Mitelman database for DHL and discovered that BCL2 is the most commonly co-rearranged gene (75%). followed by CCND1 (13%), BCL6 (10%), and BCL3 (2%).^6^ Our case was high-grade B-cell lymphoma with MYC and BCL2 rearrangement (double hit), of germinal center cell type.

Histological examination is crucial. Malignant lymphoma is characterized by a monotonous appearance of many tumor cells lacking epithelial connections and background granular lymphoglandular masses with degenerating cytoplasm. Prominent nuclear abnormalities, such as constrictions and cracks in the nucleus, are seen in tumor cells, which also exhibit finer nuclear chromatin and a considerably higher nuclear-cytoplasmic ratio than epithelial-derived cancer cells. Epithelial malignancies like lobular carcinoma and solid invasive ductal carcinoma are differential diagnoses for high-grade lymphomas like DLBCL. Granulomatous mastitis is among the possible differential diagnoses for low-grade lymphoma, which primarily consists of small- to medium-sized atypical lymphocytes.^4^ Primary differential diagnoses for breast lymphomas include DLBCL, which represents the most common subtype, follicular lymphoma, marginal zone lymphoma, mantle cell lymphoma, and chronic lymphocytic leukemia/small lymphocytic lymphoma.^7^

Surgery, radiation therapy, chemotherapy, and immunotherapy, either alone or in combination, are the mainstay of treatment of PBL. To date, there are no established treatment guidelines.^8^ There are no accepted guidelines for standardized PB-DHL treatment strategies, although combined chemotherapy and radiotherapy are presently the first-line therapeutics.^4,9^ Most studies still recommend the routine use of prophylactic CNS therapy.

Recently, it has been shown that radical mastectomy is not effective and may delay the initiation of chemotherapy. Cyclophosphamide, doxorubicin hydrochloride (hydroxydaunorubicin), vincristine sulfate (Oncovin), and prednisone (CHOP), or CHOP-like anthracycline-based chemotherapy, along with rituximab, is the standard treatment for the majority of patients with DLBCL of the breast. This may be followed by irradiation of the breast and regional lymph nodes.^10,11^ Zhang et al. conducted a multicenter cohort study of 48 patients in North-China and found that DA-EPOCH-R, alternating with high-dose methotrexate and cytarabine (DA-EPOCH-R/MA), was a promising regimen for PB-DHL, and breast irradiation gave complementary benefits for relapse reduction.^11^ Our patient was given four cycles of DA-EPOCH and showed a good response with complete resolution.

Although treatment approaches for PB-DHL continue to evolve, the role of more intensive regimens such as DA-EPOCH-R remains an important area of discussion, especially for younger or fit patients. Several retrospective studies and real-world analyses have suggested that DA-EPOCH-R may offer higher complete response and progression-free survival rates compared with rituximab, cyclophosphamide, doxorubicin, vincristine, and prednisone in cohorts with MYC and BCL2 rearrangements, reflecting the aggressive biological behavior of DHL. The continuous-infusion, dose-adjusted design of DA-EPOCH-R enhances cytotoxic exposure in rapidly proliferating MYC-driven tumors, providing a theoretical advantage over standard anthracycline-based regimens. Furthermore, current institutional practices and guideline recommendations, including those of the National Comprehensive Cancer Network, continue to list DA-EPOCH-R as a preferred option in DHL.^12^ In line with this evidence, our multidisciplinary team selected DA-EPOCH-R for this patient to maximize the likelihood of durable remission, and the patient’s complete resolution of disease supports the effectiveness of this regimen in appropriately selected cases.

## Conclusion

This extraordinary case of PB-DHL reinforces the importance of considering diverse lymphoma subtypes in breast masses. Successful management with DA-EPOCH-R/MA highlights the potential of personalized therapeutic approaches for optimal outcomes. As we continue to unveil the intricacies of breast lymphomas, this case serves as a beacon of hope for future advancements in diagnosis and treatment strategies.
